# Study of Antibody-Dependent Reactions of Mast Cells *In Vitro* and in a Model of Severe Influenza Infection in Mice

**DOI:** 10.3389/fimmu.2021.689436

**Published:** 2021-07-14

**Authors:** Andrey Mamontov, Igor Losev, Dmitrii Korzhevskii, Valeriia Guselnikova, Alexander Polevshchikov, Yulia Desheva

**Affiliations:** ^1^ Immunology Department, Federal State Budgetary Scientific Institution «Institute of Experimental Medicine», Saint Petersburg, Russia; ^2^ Virology Department, Federal State Budgetary Scientific Institution «Institute of Experimental Medicine», Saint Petersburg, Russia; ^3^ Department of General and Special Morphology, Federal State Budgetary Scientific Institution «Institute of Experimental Medicine», Saint Petersburg, Russia

**Keywords:** mast cells, influenza infection, vaccination, IgG antibodies, immune complexes

## Abstract

We investigated the reaction of mouse peritoneal mast cells (MCs) *in vitro* after IgG-containing immune complex introduction using A/H5N1 and A/H1N1pdm09 influenza viruses as antigens. The sera of immune mice served as a source of IgG antibodies. The concentration of histamine in the supernatants was determined at 4 hours after incubation with antisera and virus. We compared the contribution of MCs to the pathogenesis of post-immunization influenza infection with A/H5N1 and A/H1N1 influenza viruses in mice. The mice were immunized parenterally with inactivated viruses and challenged with lethal doses of drift A/H5N1 and A/H1N1 influenza viruses on the 14^th^ day after immunization. Simultaneously, half of the mice were injected intraperitoneally with a mixture of histamine receptor blockers (chloropyramine and quamatel). In *in vitro* experiments, the immune complex formed by A/H5N1 virus and antiserum caused a significant increase in the histamine release compared to immune serum or the virus alone. With regard to the A/H1N1 virus, such an increase was not significant. A/H1N1 immunization caused detectable HI response in mice at 12^th^ day after immunization, in contrast to the A/H5N1 virus. After challenge of A/H5N1-immunized mice, administration of antihistamines increased the survival rate by up to 90%. When infecting the A/H1N1-immunized mice, 90% of the animals were already protected from lethal infection by day 14; the administration of histamine receptor blockers did not increase survival. Histological examination of the lungs has shown that toluidine blue staining allows to estimate the degree of MC degranulation. The possibility of *in vitro* activation of murine MCs by IgG-containing immune complexes has been shown. In a model of influenza infection, it was shown that the administration of histamine receptor blockers increased survival. When the protection was formed faster due to the earlier production of HI antibodies, the administration of histamine receptor blockers did not significantly affect the course of the infection. These data allow to propose that even if there are antibody-dependent MC reactions, they can be easily stopped by the administration of histamine receptor blockers.

## Introduction

Influenza virus (the genus *Influenzavirus*, the family *Orthomyxoviridae*) infections remain an important medical and social problem with high incidence during annual influenza epidemics. Despite the improvement of vaccines and the development of vaccine prevention, severe cases of the disease are still encountered. The avian influenza A/H5N1 virus has been causing periodic outbreaks of severe infection in Southeast Asia since it was first detected in Hong Kong in 1997 (1). According to WHO, for the period from 2003 to 2019, the mortality rate from laboratory-confirmed influenza A/H5N1 was 30–66% [https://www.who.int/influenza/human_animal_interface/2020_MAY_tableH5N1.pdf?ua=1]. Avian influenza viruses of the H5 subtype not only circulate among waterfowl, but also continue to be periodically transmitted to humans (2). In this regard, the preparation of the corresponding vaccine strains will be relevant.

Influenza vaccination aimed mainly at the production of serum antibodies may have reduced protection against drift variants of viruses. In addition, infection can occur in a time period before a full-fledged immune antibody response is elicited. The effectiveness of influenza vaccination can go down when the vaccine strain does not match the epidemic virus (3). In some cases, low-affinity antibodies to the influenza virus that do not have virus-neutralizing properties may be involved in intensifying a secondary infection (4). During the 2009 influenza pandemic, the use of seasonal vaccines and the presence of antibodies against influenza A/H1N1pdm09, which do not have neutralizing properties, correlated with an increased risk of more severe influenza-like illness in infected people (5). The role of mast cells in the onset of antibody-dependent enhancement is poorly understood and underestimated.

Mast cells are specialized innate immune cells derived from the bone marrow and have been identified in all vertebrates. They have long been recognized as effector cells in allergic disorders and certain immune responses to parasites. Mast cells (MCs) are truly universal cells involved in complex immunological and non-immunological functions, providing direct effects and indirect regulation of other cells and their functioning in various biological processes (6). MCs synthesize and store cytokines in granules. Mouse MCs have been shown to produce cytokines IL-1β, IL-6 and TNF-α, a potent preformed pro-inflammatory cytokine that is also found in human MCs (7). Some of the granule components are preformed before MCs are activated (proteases, biogenic amines, proteoglycans, TNFα and IL-4 cytokines, as well as many growth factors). Another part of molecules secreted by MCs molecules is synthesized after activation of MCs (interleukins, TNFα, TGFβ, chemokines, eotaxin, lipid inflammatory mediators) (8).

Human IgG antibodies in rheumatoid arthritis and systemic lupus erythematosus, as well as diabetes and allergies, cause pathology through interaction with MCs (9). Monovalent IgG4 antigenic complexes are unable to bind and cross link Fcγ receptors (FcγR) and cannot stimulate antigen-presenting cells activation, but can bind FcγIIb receptors and cause suppression of MCs, monocytes and macrophages (10). MCs, the main effector cells in allergies, release various vasoactive substances, including histamine, SSRA (slow-reacting anaphylaxis factor), and serotonin (11). Leukotrienes, prostaglandins and platelet activating factors are synthesized by MCs activated by arachidonic acid. Cytokines, chemokines, and growth factors are synthesized *de novo* and released shortly after activation (12).

Allergic immunotherapy reduces the production of proinflammatory mediators with reduced migration of MCs in target organs (13). Different classes of FcγR are expressed on many immune effector cells and mediate various cellular responses such as macrophage phagocytosis, antibody-dependent NK- and T-cell cytotoxicity, and MC degranulation. High affinity mouse FcγRI is able to bind with high affinity only IgG2a isotype, while low affinity FcγRIII binds polymeric forms of all IgG subclasses (IgG1, IgG2a and IgG2b), except IgG3. Not so long ago, a third activating receptor FcRIV was discovered in mice, which binds immune complexes containing IgG2a and IgG2b with intermediate affinity (14).

MCs mainly express low-affinity and, under certain conditions, high-affinity IgG receptors. Mast cell survival and cytokine secretion depend on the ITAM (Immunoreceptor Tyrosine-based Activation Motif) presence in cytoplasmic tails of FcRγ-receptors, which provides for the signal transmission and cell activation (15).

Antibody-dependent enhancement (ADE) has been described for some viral infections in people with repeated illnesses or in previously vaccinated people, which is manifested by a severe course of infection, often fatal. ADE is believed to develop through the mechanism of facilitated penetration of the virus in complex with IgG antibodies and/or complement factors into cells with Fcγ and C3 receptors, which increases its infectivity and contributes to the development of a severe, life-threatening course of viral infection (16). ADE syndrome is characteristic of Dengue fever, in which it attracted attention and was first described (17).

Seasonal influenza vaccination successfully prevents the disease when the antigenic structure of vaccine strains and circulating viruses coincides. However, the positive effect of such vaccination is reduced in case of infection with the shift variants of the influenza virus, to which people have no immunity, resulting in increased severity of the infectious process and mortality when infected with the shift variants of the influenza virus, to which people have no immunity (18). In this regard, studies aimed at increasing the cross-protection of existing influenza vaccines, as well as at deciphering the mechanisms of aggravating viral infections during re-infection, are of particular relevance.

In connection with the above, the aim of the work was to test the hypothesis that MCs make a tangible negative contribution to the pathogenesis of viral infection due to the histamine secreted by them, which makes the course of the infectious process more severe. To study the role of MCs in the development of severe viral infection in immunized animals we used a model of infection with a drift variant of the influenza virus.

## Materials and Methods

### Viruses

In this study we used the following influenza viruses: A/Vietnam/1194/2004(H5N1) NIBRG-14 (National Institute for Biological Standards and Control, UK) [A/Vietnam(H5N1)] and A/Indonesia/5/2005(H5N1) IDCDC-RG2 (Centers for Disease Control and Prevention, USA) [A/Indonesia(H5N1)]. These strains were produced by reverse genetics by the National Institute of Biological Standards and Control (NIBSC, United Kingdom) and the Center for Disease Control and Prevention (USA) using Vero-certified vaccine-producing cells and laboratory protocols that take into account that the end use of the vaccine is administration to humans. The WHO Influenza Center recommends these strains for the preparation of inactivated influenza vaccines against avian influenza. Viruses contain modified H5 subtype hemagglutinin (HA) and are safe for humans. Also, we used the A/South Africa/3626/2013 (H1N1)pdm09 [A/South Africa(H1N1)pdm09] and A/New York/61/2015 (H1N1)pdm09 [A/New York(H1N1)pdm09] influenza viruses obtained from the National Institute for Biological Standards and Control (NIBSC, UK) repository. The A/California/07/2009(H1N1)pdm09 was obtained from the Institute of Experimental Medicine collection of viruses. All viruses were propagated in 10-day old chicken embryos (CE) and stored at -70°C.

### Determination of Histamine Production *In Vitro* by Mast Cells of Mouse Peritoneal Exudate

**Figure 1:**
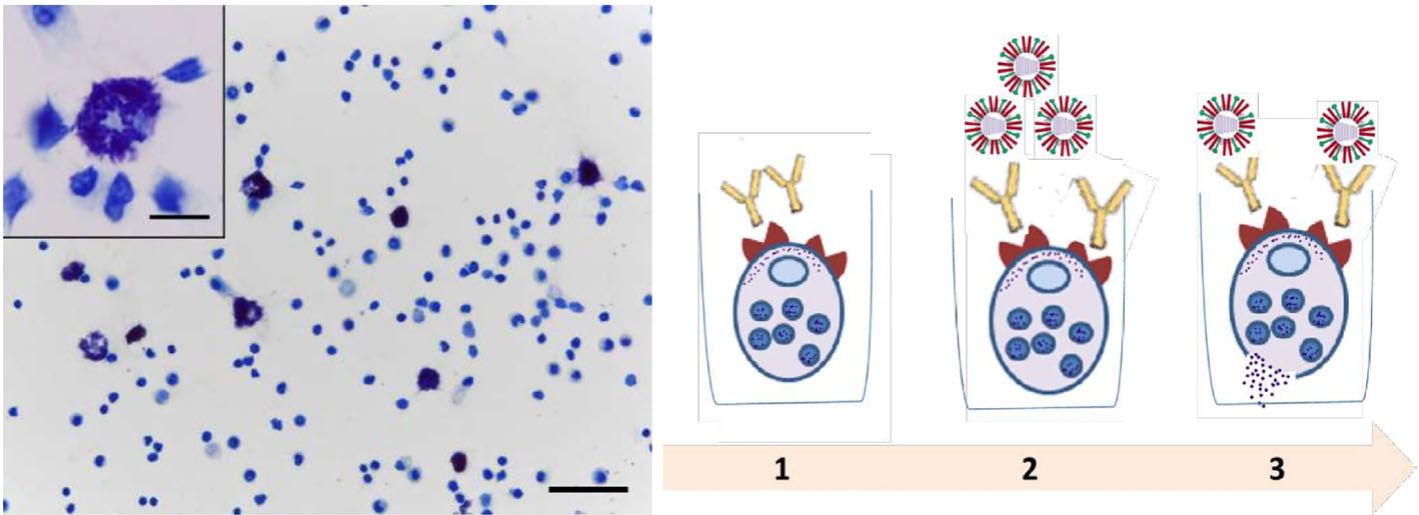
Main steps of peritoneal mast cell experiments. Peritoneal cell smear stained with toluidine blue. Mast cells are metachromatically stained purple with toluidine blue. Obj. 40x, scale bar = 50 μm; insets, obj. 100x, scale bar = 10 μm. (1) Loading of Fcγ-receptors of MC with IgG antibodies (hyperimmune serum of mice). (2) The load of MC that bound Ab using influenza viruses. (3) Degranulation and histamine release.

As a material containing MCs, we used cells from mouse peritoneal exudate. 5 ml of 0.85% phosphate-buffered saline (PBS) was injected into the abdominal cavity of the mouse, then MCs containing exudate were collected. The resulting cells were used at a concentration of 1 million cells/450 μl, among which MC content averaged at an estimated 7-10%. Influenza viruses A/Vietnam(H5N1) and A/New York(H1N1)pdm09 purified by ultracentrifugation in a 30/60 stepwise sucrose gradient were used as antigens. The source of IgG was the sera of CBA mice immunized intramuscularly (IM) with the same viruses at a dose of 20,000 hemagglutating units (HAU)/1ml in a volume of 0.1 ml. The sera were collected at day 21 after immunization and the content of antibodies to the A/Vietnam(H5N1) or A/New York(H1N1)pdm09 influenza virus in sera was confirmed by ELISA as described previously (19). Fcγ receptors of МС were loaded with IgG antibodies (incubation of peritoneal cells with hyperimmune mouse serum at a dilution of 1: 300 and 1: 900 (contact for 1 h, 4°C). Then the cells were washed from unbound antibodies and additionally loaded with antigens (influenza viruses) with a content of 5-50 HAU/1 ml (contact 1 h, 4°) C, followed by incubation for 40 min at 37° for degranulation and histamine release. The setup of the experiment on murine peritoneal cells is shown in **Figure 1**.

After the end of all incubations, results of the reaction were read with a plate modification of Shore’s method (20). The method is based on the processing of the luminescent condensation product of histamine with orthophthalic aldehyde at wavelengths 355/460 nm. The level of histamine was expressed in arbitrary units.

### Ethics Statement

The conditions for keeping animals in the vivarium provide them with a normal biological background and fully comply with the requirements of the Sanitary Rules for the Arrangement, Equipment and Maintenance of Experimental Biological Clinics (vivariums) dated 06.04.1973. The diet of animals complies with the order of the Ministry of Health No. 1179 dated 1983. The study was approved by the Local Ethics Committee for Animal Care and Use at the Institute of Experimental Medicine, Saint-Petersburg, Russia (protocol № 3/17 of 30.11.2017). Non-terminal procedures were performed under ether anesthesia inhalation. To control viral load in the lungs, animals were euthanized under ether anesthesia inhalation and cervical dislocation. The health status of the challenged mice was monitored and recorded once a day for 10-14 days post infection.

### Animals

The 8-to-10-week-old female CBA mice were provided by the laboratory-breeding nursery of the Russian Academy of Sciences (Rappolovo, Leningrad Region, Russia). Mice were maintained under standard conditions and given ten days to acclimate to the housing facility. Feeding was carried out ad libitum, in the morning with free access to water. The weight of animals at the start of the experiments was 20.0 ± 2.0 grams (mean ± SEM).

### Study of Drifted Influenza Infection in A/H5N1 and A/H1N1 Sensitized Mice

#### Immunization of Mice

Viral antigens for immunization of mice were prepared as earlier described (19). Formaldehyde-inactivated influenza viruses A/Vietnam/1194/2004(H5N1) NIBRG-14 and A/New York/61/2015 (H1N1)pdm09 or A/California/07/2009 (H1N1)pdm09 were used for IM immunization at a dose of 20,000 HAU/1ml in a volume of 0.1 ml. The control group received IM PBS as a placebo in the same volume.

#### Immunogenicity Study

Blood sera were taken from the mice on day 12 after immunization and the sera were stored at -20°C until serological tests were performed. For the hemagglutination-inhibition (HI) assay the sera were treated with receptor-destroying enzyme (RDE, Denka Seiken, Tokyo, Japan) and tested for HI antibodies against A/Vietnam/1194/2004(H5N1) NIBRG-14, A/Indonesia/5/2005(H5N1) IDCDC-RG2, A/New York/61/2015 (H1N1)pdm09, and A/South Africa/3626/2013(H1N1)pdm09 influenza viruses as previously described (19). The enzyme-linked immunosorbent assay (ELISA) was conducted to determine serum IgG antibodies against influenza viruses in 96-well microplаtes (Sarstedt AG & Co, Nümbrecht, Germany) as previously described (19). For absorption we used 20 HAU/0.1 ml of the whole purified A/H5N1 or A/H1N1 influenza viruses. As a conjugate, we used rabbit horseradish peroxidase (HPR) labeled antibodies to the Fc fragment of mouse IgG (Sigma, St. Louis, United States). The end-point ELISA titers were expressed as the highest dilution that yielded an optical density at 450 nm (OD_450_) greater than the mean OD_450_ plus 3 standard deviations of negative control wells containing conjugate only.

#### Challenge Study

On day 14 after immunization, the mice were infected intranasally with the influenza viruses at a concentration of one 50% mouse lethal dose (LD_50_) which was approximately 10^4.5^ EID_50_ for A/Indonesia(H5N1) influenza virus and 10^5^ EID_50_ for the A/South Africa(H1N1)pdm09 influenza virus. The virus was administered intranasally in a volume of 50 μl, evenly distributed between the nostrils. The LD_50_ was preliminary determined after infection with serial dilutions of virus A/Indonesia and A/South Africa from 10^2^ to 10^7^ EID_50_.

**Figure 2:**
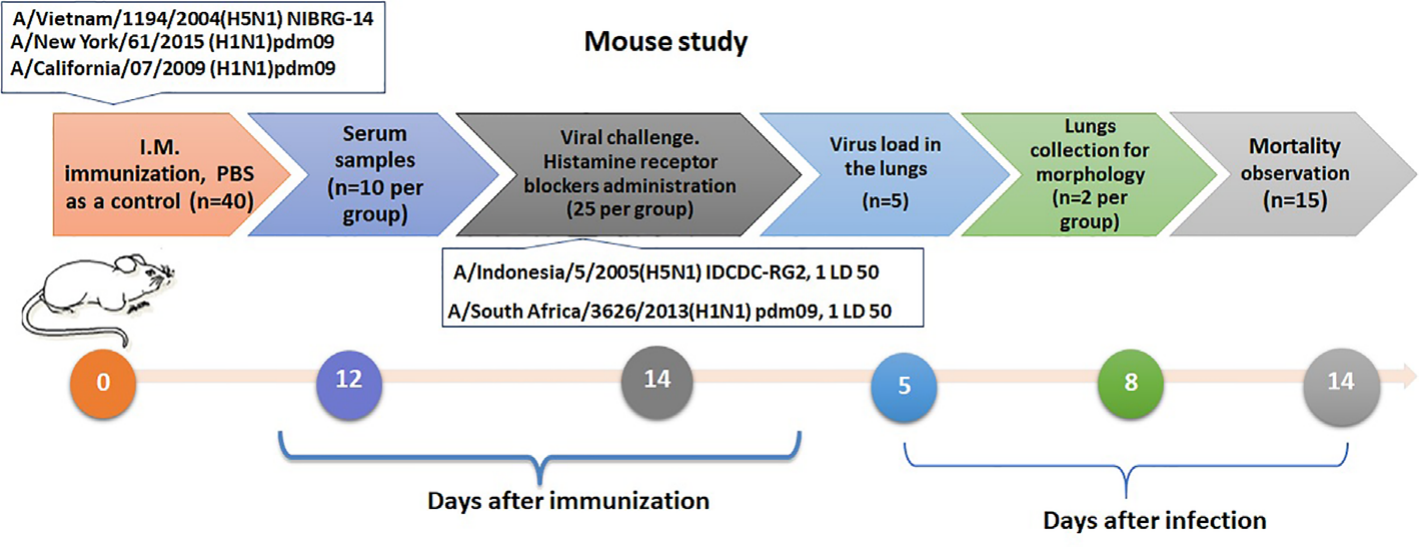
Design of an experiments to assess the role of mast cells activation by infection in influenza sensitized mice.

Simultaneously with the infection, 50% of the immune mice were administered intraperitoneally with a mixture of histamine receptor blockers H1 and H2, сhloropyramine (Egis, Guyancourt, France) and quamatel (Gedeon Richter, Budapest, Hungary), each 6.7 mg/kg body weight, in a volume of 0.1 ml, or PBS in the same volume. The experimental design is shown in **Figure 2**.

#### Evaluation of the Viral Titers in the Lungs

The lungs were collected from mice at day 5 after viral infection, homogenized using a Retsch MM-400 ball vibratory mill in PBS containing 100 U/ml penicillin, 100 μg/ml streptomycin and centrifuged for 10 min at 6000 g. Lung homogenates were titrated in developing chick embryos starting with an initial dilution of 1:10. The viral titers were calculated as a log10 of the fifty percent embryonic infectious doses (EID_50_) using hemagglutination as the endpoint, as described previously (21).

#### Histamine and Levels Measuring in the Blood Sera

In order to determine histamine levels using ELISA, blood samples were collected on the 5^th^ day after infection. Histamine production was evaluated using the EIA Histamine kit (Beckman Coulter, Brea, United States) according to the manufacturer’s instructions. All tested samples were duplicated

#### Histological Examination of the Lungs

Left- and right-lung samples from CBA mice (n = 2 per group) were used for the histological study. The samples were fixed in formalin and embedded in paraffin as previously described (22). Lung sections were stained with toluidine blue (LabPoint, Russia). Briefly, a dye solution was applied to the previously dewaxed sections and incubated for 10 min at room temperature. The sections were then rinsed, dehydrated, cleared, mounted and cover slipped.

### Statistical Processing of Results

Data was processed using Statistica software, version 6.0 (StatSoft, Inc. Tulsa, Oklahoma, USA) and graphics data were generated using Prism 8 (GraphPad software, San Diego, USA). When analyzing the results obtained, the mean values and standard deviation (M ± σ) were determined. The statistical significance of the differences was assessed using the Wilcoxon - Mann - Whitney nonparametric tests. The log-rank (Mantel–Cox test) was used to compare the survival distributions. The differences were considered significant at p <0.05.

## Results

### Estimation of Histamine Production by Mast Cells of Mouse Peritoneal Exudate *In Vitro*

**Figure 3:**
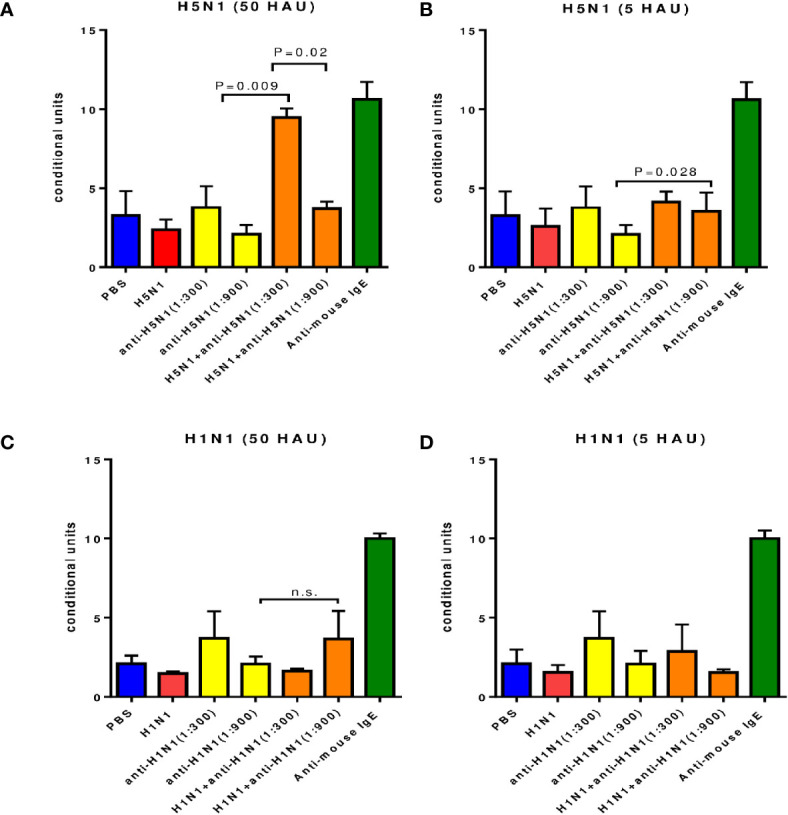
Degranulation of mast cells contained in peritoneal exudate of intact CBA mice under the influence of the immune complex. Average data from three independent experiments are presented. **(A, B)** - antibodies and influenza virus A/Vietnam/1194/2004(H5N1) NIBRG-14). **(C, D)** - antibodies a and A/New York/61/2015 (H1N1)pdm09 influenza virus); n.s., no significance.

The immune complexes formed on the cell surface by successive incubations with IgG antibodies and an antigen specific to these antibodies (A/Vietnam(H5N1) virus) with a content of 50 HAU) caused a significant increase in histamine release at serum dilutions of 1: 300 and 1: 900 (**Figure 3A**). This release of histamine was significantly higher than after incubation with IgG alone or with A/H5N1 virus alone, although it was inferior to that when exposed to mouse IgE antibodies.

While the concentration of the A/Vietnam (H5N1) virus was 5 HAU, a significant increase in the level of histamine in the culture supernatants was observed only when the A/H5N1-specific antiserum was diluted 1: 900 (**Figure 3B**).

For the A/New York(H1N1)pdm09 influenza virus, a significant increase in histamine was observed only at a virus concentration of 50 HAU and a 1:900 dilution of H1N1-specific serum (**Figure 3C**).

Thus, for both A/H5N1 and A/H1N1 viruses, there was a dose-dependent effect on virus concentration. The release of histamine was more pronounced with a higher dilution of the immune serum.

### A Model of Lethal Influenza Infection in Immunized Mice

#### Immunogenicity

**Figure 4:**
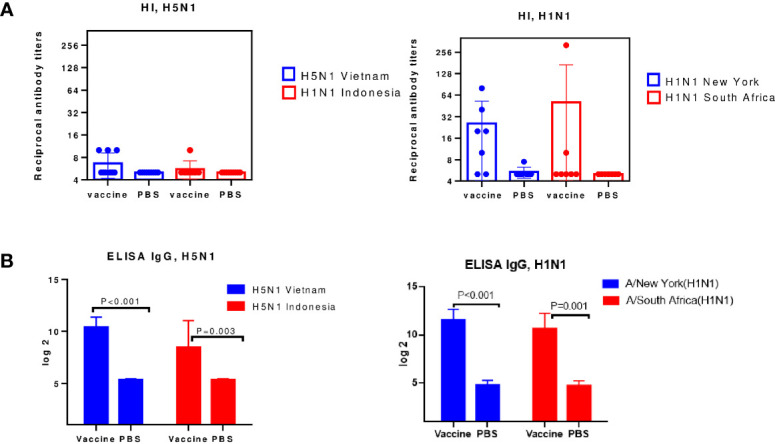
Serum antibodies on day 12 after immunization (n=8-10). Each chart shows data from one of three experiments. **(A)** The HI antibody levels against the A/H5N1 and A/H1N1 subtype. **(B)** ELISA serum IgG antibodies.

Groups of mice were administered IM with inactivated influenza viruses of A/Vietnam(H5N1) or A/New York(H1N1)pdm09 influenza viruses. Immunogenicity was assessed 12 days after immunization by the production of serum antibodies. The results of the study of virus-specific serum antibodies in the HI-test and ELISA are presented in **Figure 4**.

A **Figure 4A**, shows that on the 12^th^ day after A/H5N1 immunization, the levels of HI antibodies to homologous A/Vietnam(H5N1) and drifted A/Indonesia(H5N1) viruses were lower than 1:40, i.e. less than protective level, which is traditionally associated with at least a 50% reduction in the risk of influenza (23). HI antibodies to A/H1N1 viruses were formed at a protective level against the homologous vaccine virus and, in some cases, against the drift virus (**Figure 4A**). The levels of virus-specific IgG among immunized mice both to A/H1N1 and A/H5N1 influenza viruses significantly exceeded the levels in the control group (**Figure 4B**). Thus, viruses A/H1N1 and A/H5N1 differed in immunogenicity. When the A/New York(H1N1)pdm09 virus was administered to mice, a HI immune response was formed on the 12th day after immunization, in contrast to the A/Vietnam(H5N1) virus. These data correlate with previously obtained data on the reduced immunogenicity of A/H5N1 influenza viruses in comparison with epidemic influenza viruses (24), and may suggest that the antibodies formed as a result of A/H5N1 immunization were mainly of the non-neutralizing type.

#### Evaluation of Survival After Lethal Infection of Immunized Mice With Drifted Variant of the Same Influenza Virus Subtype

**Figure 5:**
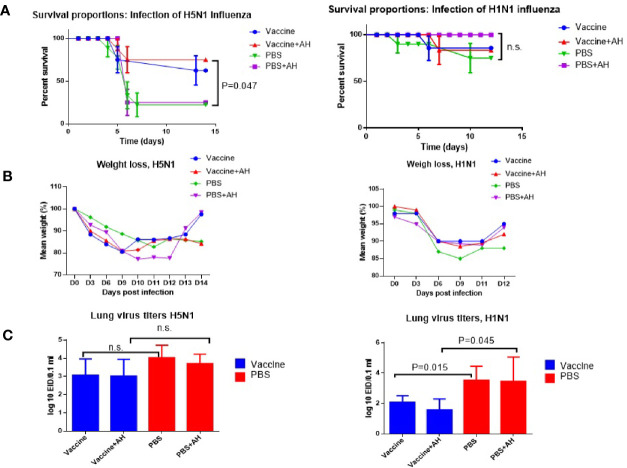
Lethal influenza infection of immunized mice. A/Vietnam/1194/2004(H5N1) immunized mice were infected with A/Indonesia/5/2005(H5N1) influenza virus. A/New York/61/2015 (H1N1)pdm09 immunized mice were infected using A/South Africa/3626/2013(H1N1)pdm09 influenza virus. AH - antihistamines. Data from one of each series of experiments are presented. **(A)** Survivals (n=20). Log-rank (Mantel-Cox) test, P – compared to PBS; n.s., no significance. **(B)** Weight loss (n=12). **(C)** Virus isolation from the lungs (n=5); n.s., no significance.

On day 14 after immunization, the mice were infected intranasally with the A/Indonesia(H5N1) or A/South Africa(H1N1)pdm09 influenza virus at a concentration of one 50% mouse lethal dose (LD_50_). Five independent experiments were carried out for the pair of A/H5N1 influenza viruses, and three independent experiments for the A/H1N1 pair. **Figure 5** shows data on mortality, weight loss, and lung virus titers.

**Figure 5A** shows that after infection of immune mice with the A/Indonesia(H5N1) virus of immune mice, when part of the mice was injected with a mixture of histamine receptor blockers, the survival rate of mice increased from 70% to 80%. The differences between vaccinated and unvaccinated animals became statistically significant. After infection with the A/South Africa(H1N1)pdm09 virus, 90% of A/New York(H1N1)pdm09 immunized mice were protected from lethal infection and the administration of antihistamines did not increase the survival rate of the mice (**Figure 5B**). Interestingly, in this case, the administration of antihistamines to non-immunized mice increased the survival rate of the mice by up to 100%, although the increase was not statistically significant. Administration of antihistamines for both A/Indonesia(H5N1) and A/South Africa(H1N1)pdm09 lethal infections did not significantly affect weight loss. **Figure 5C** shows that the isolation of infectious A/Indonesia(H5N1) viruses from the lungs of A/Vietnam(H5N1) immunized mice did not differ from those among the mice in PBS group. And in the case of the A/South Africa(H1N1)pdm09 infecting virus, the levels of the virus in the lungs differed from those among non-immunized mice, both with and without the use of histamine receptor blockers. These data show that immunization with inactivated A/H1N1 virus may cause a higher level of protection against infection with drifted variant of virus on day 14 after immunization compared to the A/H5N1 virus. This could be due to a higher level of HI antibodies to the A/H1N1 virus which had already managed to form by the 12^th^ day after IM immunization, although 3-4 weeks are considered the optimal time for the formation of a full-fledged humoral immune response to influenza vaccination (25).

### Sublethal A/South Africa(H1N1)pdm09 Influenza Infection in Immunized Mice

**Figure 6:**
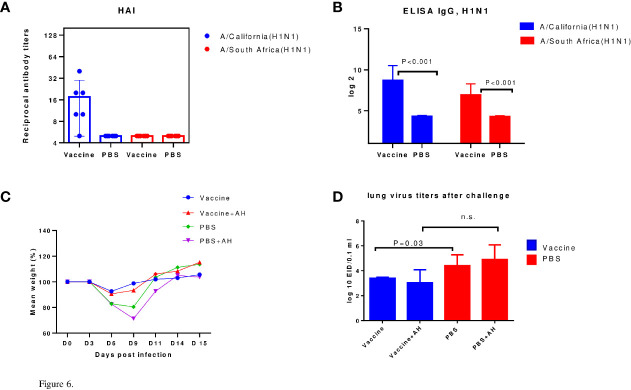
Protection against sublethal infection with drift A/H1N1pdm09 influenza virus. A/California/07/2009 (H1N1)pdm09 – immunized mice were infected using A/South Africa/3626/2013(H1N1)pdm09 influenza virus. AH - antihistamines. **(A)** The HI data (n=6). **(B)** ELISA IgG (n=6). **(C)** Weight loss dynamics (n=10). **(D)** Virus isolation from the lungs (n=5); n.s., no significance.

The above pair of viruses did not differ in the structure of hemagglutinin so significantly, since the challenge virus was isolated in 2013, and the vaccine virus was isolated in 2015. So, we tried to find out what will happen if immunization and infection with even more distant variants of the influenza A/H1N1 pandemic strain are carried out. The pandemic virus A/California (H1N1) pdm09 was first isolated in 2009 and has been included in the strains recommended for the preparation of influenza vaccines for almost 10 years. For immunization, we used the A/California (H1N1) pdm09 virus, inactivated as indicated above. Infection was carried out with virus A/South Africa at a sublethal dose, which was approximately equal to 10^4^ EID_50_. The results are shown in **Figure 6**.

Thus, it has been shown that both A/California/07/2009 (H1N1)pdm09 and A/New York/61/2015 (H1N1)pdm09 influenza viruses have a higher immunogenicity in mice than A/Vietnam/1194/2004(H5N1) NIBRG-14 influenza virus as estimated using HI assay. It was shown in models of lethal and sublethal infection of immunized mice that in A/H5N1-immized mice, mortality and weight loss were reduced after A/Indonesia/5/2005(H5N1) IDCDC-RG2 infection by histamine blockers administration but introduction of antihistamines did not affect the survival or weight loss of A/H1N1-immunized mice upon infection with the A/South Africa/3626/2013(H1N1)pdm09 virus. The use of antihistamines slightly reduced mortality among control (non-immune) animals after A/South Africa/3626/2013(H1N1)pdm09 lethal infection. Unlike A/H1N1 immunization, A/H5N1 immunization did not reduce pulmonary infection according to lung virus titers data.

### Determination of Serum Histamine Levels After Infection of Immunized Mice

**Figure 7:**
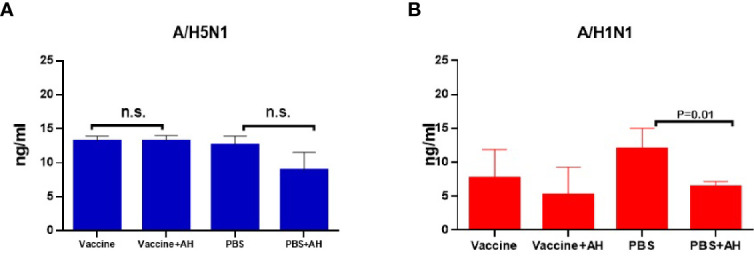
Serum histamine levels (n=5, in duplicates). **(A)** The A/Vietnam/1194/2004(H5N1) NIBRG-14 immunization followed by A/Indonesia/5/2005(H5N1) IDCDC-RG2 infection. The chart shows data from one of three independent experiments; n.s., no significance. **(B)** The A/California/07/2009 (H1N1)pdm09 immunization followed by A/South Africa/3626/2013(H1N1)pdm09 infection.

**Figure 7** shows that histamine levels in the sera of immunized mice were not affected by administration of antihistamines during infection with the A/Indonesia/5/2005(H5N1) IDCDC-RG2 influenza virus, and slightly decreased in the case of infection with this virus in non-immune mice, although the differences were not statistically significant. Administration of antihistamines led to a decrease in serum histamine levels only among non-immune mice following infection with the A/South Africa/3626/2013(H1N1)pdm09 virus (P=0.02).

### Histological Examination of Lung Tissue

#### A/Indonesia/5/2005(H5N1) Influenza Virus Infection of A/Vietnam/1194/2004(H5N1)- Immunized Mice

**Figure 8:**
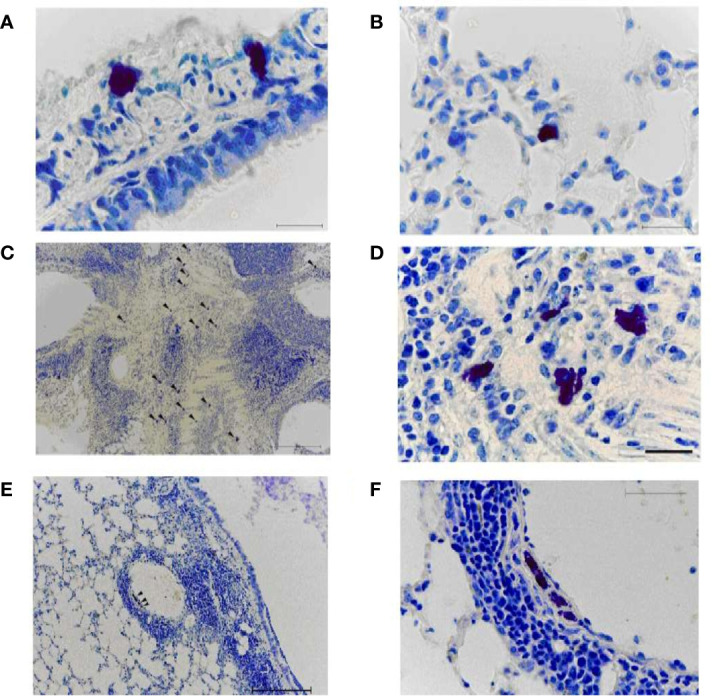
Mast cells in the lungs of vaccinated mice on day 8 after infection with A/Indonesia/5/2005(H5N1) IDCDC-RG2 influenza virus. A, **(B)** The lungs from mock-immunized mice on day 8 after infection with A/Indonesia/5/2005(H5N1) IDCDC-RG2 influenza virus. **C–F**. The lungs from A/Vietnam/1194/2004(H5N1) NIBRG-14¬-immunized mice. The arrowhead indicates a mast cell. Obj. 10x **(C, E)**, 40x **(F)**, and 100x **(A, B, D)**; scale bar = 200 μm **(C, E)**, 50 μm **(F)**, and 20 μm **(A, B, D)**.

MCs were identified in lung tissue on day 8 after immunization (**Figure 8**). When using toluidine blue, MCs in the mouse lung are stained metachromatically to a rich purple color. Due to the intense staining, MCs can already be identified at a low microscope magnification (x10). This greatly facilitates the quantitative analysis of MCs. The MC cytoplasm contains a large number of densely packed metachromatically stained granules, often shielding the nucleus. Several of lung MCs showed signs of degranulation such as extracellular granules. In this case, the granule boundaries are visible. This makes it possible to identify the different stages of MC degranulation (from weak degranulation with the secretion of single granules into the extracellular space to strong degranulation with rupture of the plasma membrane and secretion of all granules) and to assess the MC degranulation degree.

On day 8 after A/H5N1 infection of non-immune mice, rare non-activated (intact) mast cells were found in the walls of the large bronchi (**Figure 8A**) and in the alveolar septa (**Figure 8B**).

After A/H5N1 infection of the immunized mice, the enrichment of lung MCs was observed (**Figures 8C, D**, arrowhead). MCs were typically found in close proximity to both inflamed bronchi and blood vessels (**Figures 8E, F**, arrowhead). Intact MCs, as well as MCs with mild/moderate degree of degranulation were identified.

#### Infection of A/California/07/2009 (H1N1)pdm09-Immunized Mice With the A/South Africa/3626/2013(H1N1)pdm09 Influenza Virus

**Figure 9:**
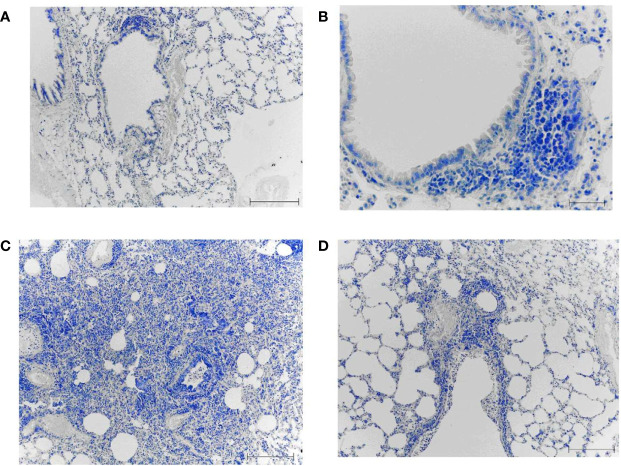
The lungs from non-immunized mice on day 8 after infection with A/South Africa/3626/2013(H1N1)pdm09 influenza virus. A, **(B)** Intact non-immunized non-infected mice. С, **(D)** The PBS-immunized mice infected with A/South Africa/3626/2013(H1N1)pdm09 influenza virus. Obj. 10x **(A, C, D)** and 40x **(B)**; scale bar = 200 μm **(A, C, D)** and 50 μm **(B)**.

The lungs of intact mice (non-immunized and non-infected) had a normal histological structure (**Figure 9A**). In some airway walls, the bronchus-associated lymphoid tissue was found (**Figure 9B**).

In the PBS-immunized A/South Africa/(H1N1)pdm09–infected group, a focal pneumonia was detected (**Figure 9C**). Large areas of atelectasis and emphysema, extensive mononuclear infiltrates in the walls of the airways and blood vessels, interstitial infiltration with thickening of the interalveolar septa were detected (**Figure 9D**).

**Figure 10:**
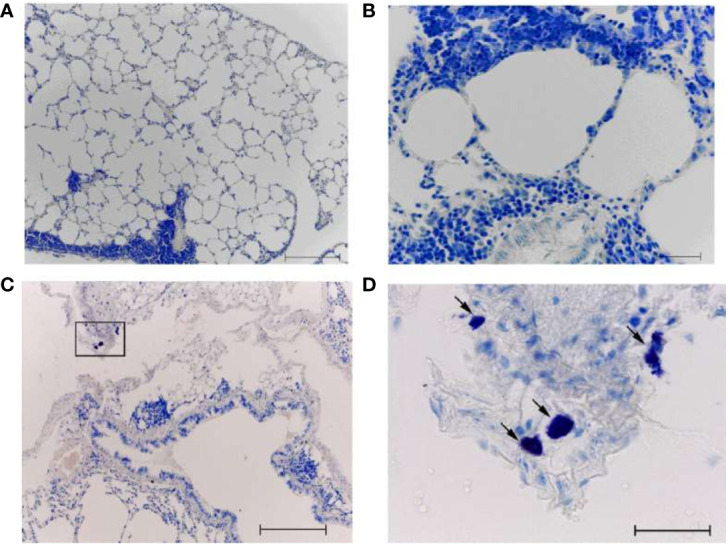
The lungs of A/California/07/2009 (H1N1)pdm09–immunized mice on day 8 after infection with A/South Africa/3626/2013(H1N1)pdm influenza virus. With **(C, D)** and without **(A, B)** antihistamine treatment. Black frame - the area of mast cell localization (D - the same area at high magnification). The arrow indicates a mast cell. Obj. 10x **(A, C)** and 40x (B, D); scale bar = 200 μm **(A, C)** and 50 μm **(B, D)**.

After A/H1N1–immunized animals were infected with A/South Africa/3626/2013(H1N1)pdm09 infection of AH1N1–immunized animals, extensive mononuclear infiltrates were present in the airway walls (in particular, along their entire length) and around the blood vessels (**Figures 10A, B**). Dystrophic changes in the ciliated epithelium and desquamation of epithelial cells in the bronchi were revealed. Some signs of diffuse alveolar damage including interstitial infiltration with thickening of the interalveolar septa and emphysema were observed. At the same time, when antihistamines were administered simultaneously with infection to the A/H1N1–immunized animals (**Figures 10C, D**), the lung tissue looks the same as in the intact control. Extensive mononuclear infiltrates, dystrophic changes and desquamation of the epithelium were not revealed. No signs of alveolar damage were found. In some airway walls, the presence of bronchus-associated lymphoid tissue was noted (**Figure 10C**). Intact MCs were also identified (**Figure 10D**, arrow).

## Discussion

Previously, it has been noted in mouse macrophage cell lines that antibodies against influenza virus hemagglutinin (HA) or neuraminidase (NA), the two main structural components of the viral envelope, were able to induce ADE (18, 26). The enhancing activity was strain-dependent and FcR-mediated (27–29).

Our *in vitro* experiments were carried out not with a purified population of MCs, but with a pool of peritoneal cells, in which the proportion of MCs is about 10% relative to other cell types (lymphocytes, macrophages, neutrophils, eosinophils). MCs are the main cells with an immediate release of histamine. This suggests that the response to the immune complex in our experiments in the form of a histamine release reaction was predominantly in response to the action of MCs.

The A/Vietnam(H5N1) and A/New York(H1N1)pdm09 strains were used as antigens for the formation of immune complexes on antibody-loaded MCs from peritoneal exudate of mice, and for both viruses, the dose of 50 HAU was more effective in stimulating the production of histamine compared to 5 HAU (**Figure 3**). The opposite trend was observed with respect to IgG containing sera when a higher dilution of immune sera caused a higher production of histamine. Thus, in our *in vitro* study of histamine production under the influence of IgG-containing immune complexes, a dose-dependent effect on the concentration of the virus was demonstrated.

Interestingly, in previous studies of antibody-dependent reactions in SARS-Cov-1 *in vitro* it was shown that the more diluted anti-S mouse sera exhibited significantly greater SARS CoV ADE effects on HL-CZ cells compared to less-diluted mouse sera (30).

As is generally known, MCs are the main source of histamine in the body. To block the action of histamine in experiments on mice, we used a mixture of H1 and H2 blockers (although the main type of receptors on cells responsible for the anaphylactogenic effects of histamine are H1 receptors). Our previous data indicate that the introduction of histamine receptor blockers decreased in lethality after A/H5N1 infection of vaccinated mice (31). In the present study, it was shown that after challenge with the A/H1N1 virus, protection reached 90% of the immunized animals and the administration of antihistamines did not make an additional contribution to improving survival. But as estimated by the histological features in the lungs, antihistamines still had a positive effect, since when they were administered, the signs of pneumonia decreased (**Figures 10C, D**). At the same time, the administration of antihistamines to non-immunized mice also facilitated the course of A/H1N1 influenza infection.

The data obtained in our study correlates with earlier studies. Thus, a positive effect of antihistamines on A/H5N1 infection in mice has been demonstrated previously (32). The effect of vaccination on the course of the subsequent influenza infection was also previously studied in animal experiments. The C57BL/6J mice received two doses of subunit vaccine IM 4 weeks apart. One month later, the animals were challenged with a sublethal dose of the A/Hong Kong/2/1968(H3N2) strain. Vaccinated mice showed no clinical symptoms of disease, while unvaccinated animals lost weight from 3 to 7 days after vaccination, but subsequently recovered. After infection with the A/H5N1 virus, unvaccinated animals showed moderate signs of illness for 7 days, after which they recovered. On the contrary, the vaccinated mice developed an infection, accompanied by a critical loss of body weight by 6-8 days after infection, which indicates the formation of immunopathology (33).

Currently, there is a widespread tendency to develop universal influenza vaccines with a wide spectrum of action (34). The induction of a wide range of cross-reactive immunoglobulins is fraught with the risk of an antibody-dependent enhancement of the infectious process, when antibodies that do not have neutralizing activity facilitate the penetration of the virus into cells due to FcγR-dependent endocytosis. Antibodies to M2e have low neutralizing activity, however, they provide protection through FcγR-mediated mechanisms, in particular, through antibody-dependent phagocytosis of viral particles. The formation of an immune response to a limited set of antigenic determinants of the influenza virus does not exclude the emergence of escape mutants, to which the body will not have immunity, which can lead to a large number of serious diseases. An example is previously reviewed cases of increased vulnerability to pandemic A/H1N1 strains among people who received seasonal influenza vaccine (33).

Previously, it was shown that monoclonal Abs that bound the globular head or base of the head domain of influenza HA may induced destabilization of the HA stem domain to increase infection of the macrophage-like cell line in an Fc-dependent manner. Non-neutralizing monoclonal Abs may cause enhanced respiratory disease in mice following A/Hong Kong/1/1968 (H3N2) influenza virus challenge (35). Therefore, *in vitro* tests and animal models need to be developed to confirm preclinical safety of next-generation influenza vaccines that may elicit antibodies which do not block influenza virus–receptor interaction.

## Conclusions

When vaccinated with inactivated influenza vaccines, serum antibodies are the main protective component. There is a gap between vaccination and the formation of a full-fledged antibody response. In the case of viruses with H5 hemagglutinin, the immune response may occur later than in the case of immunization with seasonal influenza viruses. Non-neutralizing antibodies can interact with MCs when infected with the avian influenza virus. Our data on the positive effect of histamine receptor blockers on the course of post-vaccination infection in drifted influenza viruses can help to overcome unwanted effects. The data obtained can be useful in the implementation of vaccines against other viral infections.

## Data Availability Statement

The raw data supporting the conclusions of this article will be made available by the authors, without undue reservation.

## Ethics Statement

The animal study was reviewed and approved by The Local Ethics Committee for Animal Care and Use at the Institute of Experimental Medicine, Saint-Petersburg, Russia (protocol № 3/17 of 30.11.2017).

## Author Contributions

AM – *in vitro* experiments, mouse model, data analysis, manuscript preparation. IL – mouse model, serology, ELISA test, data analysis. DK – histological examination, data analysis, manuscript editing. VG –lung preparations, histological examination, manuscript editing. AP - general leadership, data analysis, manuscript editing. YD - study design, data curation, manuscript preparation, final editing. All authors contributed to the article and approved the submitted version.

## Funding

This work was supported by the Federal State Budgetary Scientific Institution «Institute of Experimental Medicine»

## Conflict of Interest

The authors declare that the research was conducted in the absence of any commercial or financial relationships that could be construed as a potential conflict of interest.

## Abbreviations

ADE, Antibody-dependent enhancement; AH, antihistamines; CE, chicken embryos; EID_50_, fifty percent embryonic infectious doses; ELISA, enzyme-linked immunosorbent assay; FcγR, Fcγ receptors; HA, hemagglutinin; HAU, hemagglutinating units; HI - hemagglutination inhibition reaction; HRP, horseradish peroxidase; IM, intramuscularly; ITAM, Immunoreceptor Tyrosine-based Activation Motif; LD_50_, 50% mouse lethal dose; MCs, mast cells; NA, neuraminidase; PBS, phosphate-buffered saline; VIP, vasoactive intestinal peptide.
